# High-Density Bio-PE and Pozzolan Based Composites: Formulation and Prototype Design for Control of Low Water Flow

**DOI:** 10.3390/polym13121908

**Published:** 2021-06-08

**Authors:** Nicola Schiavone, Vincent Verney, Haroutioun Askanian

**Affiliations:** CNRS, Clermont Auvergne INP, ICCF, Université Clermont Auvergne, F-63000 Clermont-Ferrand, France; nicola.schiavone@sigma-clermont.fr (N.S.); vincent.verney@uca.fr (V.V.)

**Keywords:** 3D printing, bio-based polyethylene composite, X-ray tomography

## Abstract

An eco-friendly solution to produce new material for the material extrusion process is to use quarry waste as filler for biopolymer composites. A quarry waste that is still studied little as a filler for polymer composites is pozzolan. In this study, the optimization of the formulations and processing parameters of composites produced with pozzolan and bio-based polyethylene for 3D printing technology was performed. Furthermore, a precision irrigation system in the form of a drip watering cup was designed, printed, and characterized. The results showed that the presence of the pozzolan acted as a reinforcement for the composite material and improved the cohesion between the layers of the 3D printed objects. Furthermore, the optimization of the process conditions made it possible to print pieces of complex geometry and permeable parts for the control of the water flow rates with an order of magnitude in the range from mL/h to mL/day.

## 1. Introduction

The study and development of the material extrusion process as an additive manufacturing technique has never stopped since the first version presented by Scott Crump at Stratasys Inc. in 1989 [1]. Due to its low cost, ease of use, and the possibility to produce very complex customizable parts, the material extrusion process has consolidated its presence in the manufacturing market [2].

There are many domains where additive manufacturing can be used, such as aeronautics, automotive and medical applications. Among these domains, the field of agriculture equipment has a lot of potential, and few studies have been conducted in relation to it. In this field, precision irrigation (a subgroup of precision agriculture) creates and optimizes systems to control the irrigation of the plants, depending on the environmental and operational context, and to avoid water and energy waste [3,4,5,6,7,8,9,10].

In terms of 3D printing filament materials, acrylonitrile butadiene styrene (ABS) and polylactic acid (PLA) are the polymers filaments that dominate the market of 3D printing due to their availability and suitability from the point of view of adequate mechanical properties and dimensional accuracy of the final printed object [11,12]. Other common polymer filaments used for 3D printing fabrication are polyamide (PA), high-impact polystyrene (HIPS), polycarbonate (PC), and polyether ether ketone (PEEK), which are adopted for final application where higher mechanical properties and thermal stability are needed [13,14,15,16].

Compared to common polymer filaments for 3D printing, a polymeric matrix with competitive properties such as excellent impact properties, high chemical stability and excellent electrical insulation is the polyethylene (PE) matrix [17].

While PE dominated the polymers market with a share of 25.7% in 2019, and despite the great interest by both industries and researchers in improving the performance and functionality of 3D printed objects, there is a lack of information about the 3D printing of polyethylene, with only a few studies conducted on polyethylene used as filaments for the material extrusion process [18,19,20,21,22,23]. An interesting grade of PE that can be used as a 3D printing polymer filament is bio-based polyethylene (BioPE). This type of polyethylene can be synthesized from biomass (e.g., sugar cane, sugar beet and wheat grain). Its chemical structure is identical to petroleum-based polyethylene. It is industrially available and has a great chance to be used for eco-friendly future plastic products [24]. The characteristics of polymer materials which are required for proper 3D printing can be summarized as follows: sufficient stiffness of the filament to avoid Euler buckling, a melting temperature below the upper limit of the 3D printing extruder, adequate thermorheological properties to maintain the shape during cooling and having optimal cohesion between the layers of the printed parts [25]. Concerning these aspects, polyethylene presents some issues regarding suitability for the material extrusion process. For example, as a semi-crystalline polymer, PE is characterized by a strong volume change during cooling, which can lead to warpage of the final object and losing the correct shape with weak interlayer welding. Moreover, this polymer has low adhesion with most of the materials that characterize the printer beds, causing detachment of the object from the printer bed during printing [26,27,28]. Consequently, it is still a challenge to properly print a 3D final object with polyethylene, and more knowledge on the optimal printing conditions is needed to obtain a comparable final piece as a common 3D printing polymer filament. A possible way to improve the dimensional accuracy and welding layers of printed parts is the use of inorganic fillers by developing new composites. The presence of the fillers affects the viscoelastic behavior and thermal expansion, reducing the mobility of the polymeric chains during cooling [29]. The optimization of a polymer composite also has the objective of maximizing the filler content, limiting the use of the polymeric matrix and therefore of plastic waste. However, the use of a composite with a high filler content makes it challenging for the extrusion process to obtain a uniform diameter filament and for the printing, since it is possible for the nozzle to become clogged [30,31,32].

Pozzolan powder is an inorganic filler not commonly used for polymer composites. It is a pyroclastic rock extensively used in different sectors such as construction, buildings and roads, sanitation, and agriculture. In the agriculture field, pozzolan is used for drainage, soil amendments, substrate crops, and the restoration of soil. This rock has a natural or artificial origin, and the composition is rich in silicon dioxide and aluminum oxide. It has a porous form and high thermal stability [33,34,35,36,37].

The pozzolan separation process generates a huge amount of a lateral fine fraction of pozzolan. The generated quantity could achieve 50% of the total treated pozzolan mass. Most generated fine fractions are considered by-products and have a very limited market. Each year, there is an accumulation of many thousand tons in each carrier [38]. At the same time, the directives of the quarries department specify that all the extracted pozzolan must be destined for a specific use [39]. Currently, for environmental issues, the world is heading toward zero-waste production in every field and zero or even a positive environmental impact. Pozzolan is a nontoxic material, and it could be used as a fertilizer in soil [40]. Consequently, the valorization of this by-product as composite filler can be a solution to developing new eco-friendly materials. In this domain, only a few studies have focused on the valorization of natural inorganic material by-products as filler for bio-based polymers intended for 3D printing [35,41,42,43].

In this work, a new composite based on high-density biopolyethylene and pozzolan by-product is studied. Four formulations are produced in the form of 3D printing filaments for the material extrusion process and analyzed through thermal and rheological characterization. Subsequently, the obtained composite filaments are used for the printing of mechanical specimens and the printing of a drip watering close system prototype. The prototype is characterized by X-ray tomography for morphological analysis, and the results are compared with the water flow measurements obtained by a simulation test in real conditions.

## 2. Materials and Methods

### 2.1. Raw Materials

Pozzolan waste was obtained from Pouzzolanes des domes S.A.S (Le Vauriat, St Ours des Roches, France). It is an industrial by-product and, in accordance with the Total Alkali Silica (TAS) classification, was placed at the lower limit of trachybasalts [39,40]. The powder was red pozzolan and was part of storage on the quarry site. It was obtained by process separation after heating for 15 min at a temperature of 600 °C. The De Brouckere mean diameter of the powder was 56 µm, and the specific surface was 2939 cm^2^/g. The bio-based bimodal high-density polyethylene (HDPE) SGE 7252 was used as a polymer matrix and purchased by Braskem (São Paulo, Brazil). The density and melt flow index (190 °C/2.16 kg) values were 0.952 g/cm^3^ and 2 g/10 min, respectively.

### 2.2. Preparation of Composite Pellets and the Filaments for 3D Printing

**Figure 1:**
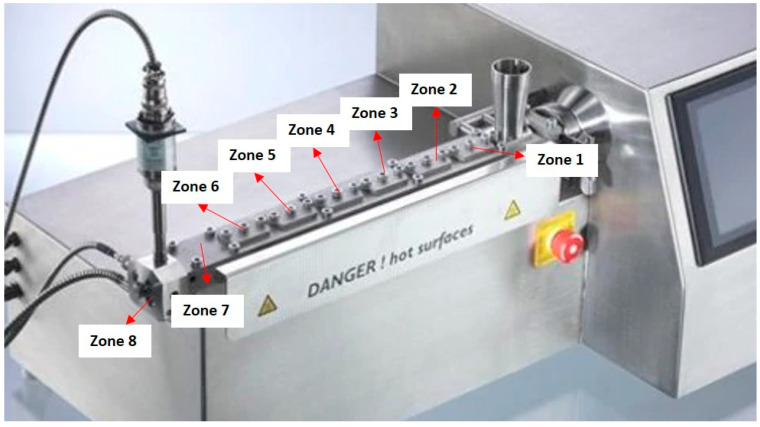
Schematic illustration of the extruder zones.

The preparation of composite pellets and 3D printing filaments was performed with a Thermo Fisher Scientific Pharma 11 twin-screw extruder (7 heat/cool zones plus 1 heating zone for the die; L/D ratio = 40:1) (Figure 1) (Waltham, MA, USA). The process conditions are reported in Table 1. For production of the composites pellets, the feed for the pozzolan was placed between Zones 6 and 7 of the extruder, and the mass flow rate was set up to attain different mass ratios of pozzolan equal to 0%, 20%, 40%, and 60%. To produce a 3D printing filament with a regular diameter of 1.75 mm, a second extrusion was performed using the composite pellets obtained previously. A second extrusion was performed in order to avoid a second feeding (powder feeding), which would lead to flow instability and less regularity of the polymeric melt at the exit of the extrusion die. The sample code was resin type-%PR, where %PR was the value of the pozzolan mass percentage.

### 2.3. Thermal Characterization

To verify the amount of pozzolan and analyze the effect of the fillers on the thermal stability, thermogravimetric analysis was carried out for all samples. For this purpose, a PerkinElmer TGA 4000 (Waltham, MA, USA) in the range from 25 to 600 °C under an N_2_ atmosphere at a rate of 10 °C/min was utilized.

The crystallinity and thermal behavior of the samples studied were investigated by a METTLER TOLEDO DSC 3+ differential scanning calorimetry (Columbus, OH, USA) under a nitrogen atmosphere. The DSC thermograms were obtained by using 8–10 mg of material for each sample. A heat–cool–heat cycle experimental method was, used and the first and second heating stages were set from room temperature to 180 °C at 10 °C/min, while the cooling stage went from 180 °C to room temperature at 10 °C/min. The percentage of crystallinity *X_c_* of the samples was calculated using the following equation:

*X_c_* = (Δ*H_m_* − Δ*H_cc_*)/(Δ*H*_*m*0_ − (*w/w*)*_p_*) × *100* (1)

where Δ*H_m_*, Δ*H_cc_*, Δ*H_m_*_0_, and (*w*/*w*)*_p_* are the melting enthalpy, the cold crystallization enthalpy, the melting enthalpy of pure crystalline polymers, and the mass fraction of the polymer in the matrix, respectively. The Δ*H_m_*_0_ taken was 286.7 J/g for the HDPE [44].

In order to compare the crystallization kinetics of the composites as a function of the pozzolan contents, Avrami analysis was performed, and the half-crystallization time (*t*_1/2_) was measured for each sample. The thermal cycle was as follows: heating from 30 °C to 180 °C at 40 °C/min, remaining for 3 min at 180 °C to uniform the temperature in the sample, and then cooling down to 118 °C (crystallization temperature) at 40 °C/min [45].

### 2.4. Rheological Measurements

The loss viscosity (*η*′) and storage viscosity (*η*″) of the melted composites were measured using a shear dynamic experiment. The tests were carried out with an ARES rheometer manufactured by TA Instruments/Waters Corporation (Milford, MA, USA). The frequency sweeps method was chosen, using parallel plate geometry with a diameter of 8 mm and a gap height of 1 mm. The deformation value was chosen following the verification of the linear viscoelasticity range, which was performed with the strain sweep method at a frequency of 10 rad/s. The temperatures used for the test were 140 °C, 150 °C, 170 °C, and 180 °C, while the strain and frequency range were set at 10% and between 0.1 and 100 rad/s, respectively. The Newtonian zero shear viscosity *η*_0_ could be determined from the extrapolation of the arc of a circle plotted from the experimental data, which is characteristic for a Cole–Cole distribution [46].

The effect of the pozzolan on the volumetric contraction stress of the composites was analyzed with the force gap measurement as a function of the temperature and time with a force gap test in a sequential configuration. The gap was fixed at 1 mm, and the temperature profile was characterized by a series of steps of 5 °C each. The initial and final temperatures were 200 °C and 100 °C, respectively, and a duration of 2 min was set for each step [47].

### 2.5. 3D-Printed Specimens and Drip Watering Close System Prototype Preparation

The 3D printing filament composites were used for printing the specimens for the mechanical tests and the prototypes of close watering systems. The printer used was the Prusa i3 MK3S (Prague, Czech Republic), and the g-code files were elaborated through PrusaSliser software. The geometry of the specimens was selected according to an ASTM D638 for the tensile test and an ASTM D256 for the Charpy impact test. All the specimens were printed on a polypropylene plate. The filling rate and the infill pattern used for the mechanical test samples were set to 100% and linear at an angle of ±45° to the longitudinal axis, respectively. The drip watering prototypes were printed starting from the models developed with the 3D CAD software Autodesk Fusion 360 (Mill Valley, CA, USA). The system was conceived as a bottle cap having a geometrical structure that facilitated insertion into the soil, anchoring the system. Moreover, a permeable porous structure was obtained by using a gyroid 3D printing infill pattern without walls. For the analysis of the porous structure effect on the liquid water flow, three print sets with different infill densities (IDs) of the infill pattern were used, which were equal to 50%, 60%, and 70%.

**Figure 2:**
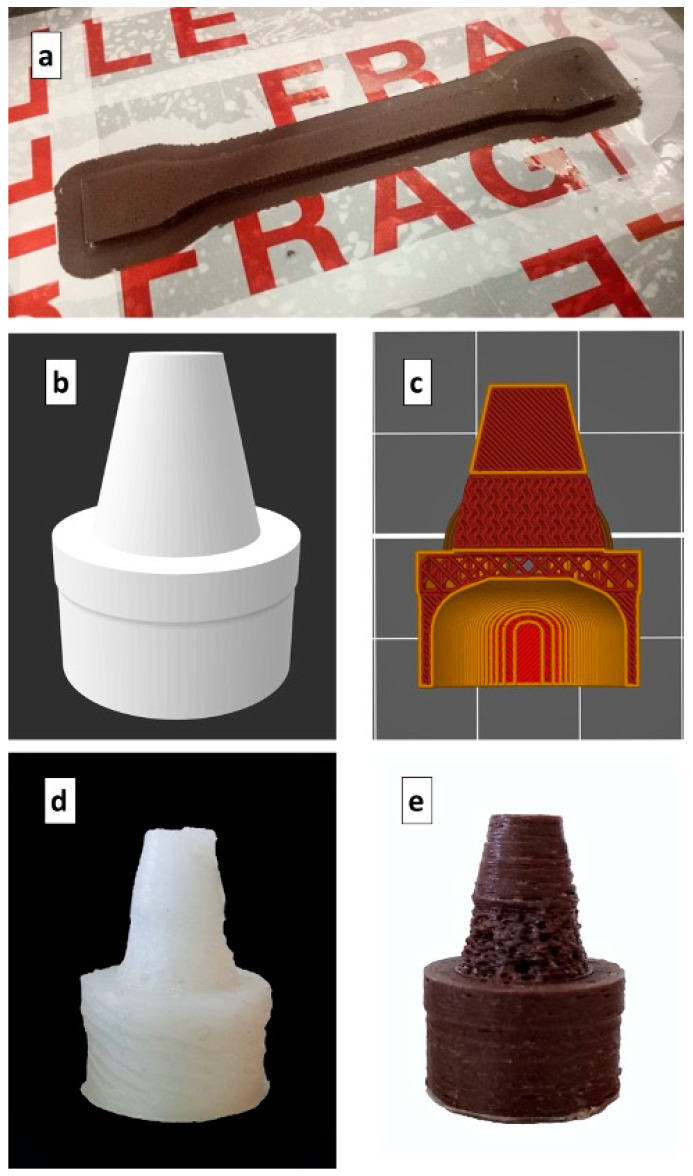
(**a**) Tensile specimen printed with the pozzolan composite. (**b**) 3D model of the prototype for precision irrigation. (**c**) Longitudinal section of the sliced prototype. (**d**) Prototype sample 3D printed with neat HDPE. (**e**) Prototype sample 3D printed with composite at 20% pozzolan.

The printing temperature of the nozzle used in this work was identified to be in the range of 250–270 °C. The used temperature value was set to ensure a suitable viscosity for the extrusion of the HDPE composites and to avoid the material clogging at the nozzle. Polyethylene required particular attention due to the poor adhesion with the materials used for the commercial printer plates and the evident shrinkage during cooling. An atactic polypropylene film was used as a printer bed in order to ensure adhesion with the printed HDPE through the interdiffusion of macromolecular chains, which occurs mainly in the amorphous phase [48,49]. A 3D-printed specimen is shown in Figure 2a.

Another aspect to consider was the cooling rate of the material for the solidification control during printing. The printing of small parts is a critical point, since the added new layers may not have sufficient time to solidify and retain their shape. In the case of HDPE composites, due to the low glass transition temperature, to reach an adequate viscosity of the deposited material, it is important to reach temperatures near crystallization rapidly; otherwise, the geometry is not preserved. In terms of process conditions, the use of an air fan can control the cooling of the material by setting a correct fan rotation speed. For the samples produced in this work, a fan was set 0% of its maximum speed for the first 20 layers and 90% for the rest of the layers.

The 3D model of the system is shown in Figure 2b, and the process conditions used for printing are reported in Table 2.

### 2.6. Mechanical Characterization

The tensile properties were evaluated according to an ASTM D638 IV (West Conshohocken, PA, USA), using a Lloyd EZ50 mechanical test machine (Bognor Regis, UK) at a cross-head speed of 30 mm/min. Tests were carried out at room temperature, and at least five specimens were tested for each sample. The size of the specimens was 115 ± 0.2 mm in length, 10 ± 0.1 mm wide, and 4 ± 0.05 mm thick. The impact properties, according to an ASTM D256 (West Conshohocken, PA, USA)) using Zwick/Roell HIT pendulum impact testers (Ulma, Germany) with a pendulum of 50J in the Charpy configuration, were also evaluated. The size of the impact specimens was 55 ± 0.1 mm in length, 10 ± 0.1 mm wide, and 4 ± 0.06 mm thick. All the results were averaged to obtain a mean value.

### 2.7. Characterization of the Prototype System

#### 2.7.1. X-ray Tomography

To analyze the morphological and lactic structure of the printed prototype, a Skycan 1174 (Edinburgh, UK) was used for X-ray radiography and tomography. Each sample was placed on a rotating plate while the X-ray beam passed through. The images were recorded by a CCD camera with a resolution of 1024 × 1024 pixels, which revealed the different levels of X-ray absorption of the sample. The total exposure time for each sample was 450 s, and the pixel size was 29.7 µm. Two images were taken per angular position and were averaged. After the reconstruction of the 3D structure part, CT analysis software was used to measure the total porosity of the internal section of the permeable part of the drip watering prototype. Furthermore, the isometric projections were obtained using DataViewer software.

#### 2.7.2. Measurement and Analysis of the Water Flow in Real Conditions

##### Water Flow Measurement System

**Figure 3:**
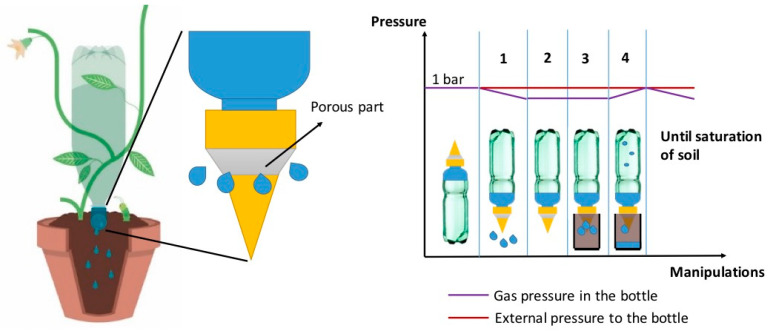
Simplified diagram of the operating principle of the drip watering system.

The measurement of the water flow rate of the drip watering prototype was carried out with the systems shown in Figure 3. The system ias characterized by the prototype of a watering cap placed on a PET bottle filled with 0.5 L of water and a beaker containing commercial soil. The adopted experimental method consisted of weighing the beaker/soil system at different times, following the passage of water from the bottle to the soil through the permeable volume of the prototype. The bottle/prototype system was maintained by a fixed support, while the beaker/soil system was placed on a mobile support so as to not manipulate the bottle and modify the internal pressure. Each measurement was made using a volume of soil equal to 90 mL, and this was repeated three times. The saturation point of the soil with a volume of 90 mL was equal to 30 ± 2 mL of water.

##### Analysis of the Water Flow through the Prototype System

A mathematical model was developed to quantitatively compare the results obtained by the water flow measurement. However, this paper goes beyond a detailed study of the fluid dynamics of the considered prototype. For this reason, simplifying hypotheses were considered, which will be explained in this paragraph.

The total flow of the water from the bottle to the soil was defined by considering a mass conservation balance.

The mass balance was simplified by taking into account only the resistance to water transport due to the permeable part of the prototype. The liquid water flow can be described as [50]

*d*(*ρ V*)/*d*(*t*) = *ρ Q_D_* (2)

where *d*(*ρ V*)/*d*(*t*) is the transitory term related to the accumulation of water in the volume of the soil, *ρ* is the density of the water (kg/m^3^), *V* is the volume of water absorbed by the soil (m^3^), and *Q_D_* is the volumetric flow rates of water through the permeable part of the prototype.

In particular, considering the laminar flow condition and the hypotheses on single-phase fluid flow, the term *Q_D_* can be described by Darcy’s law [51]:

*Q_D_* = (*ρ k A g* Δ*h*)/(*µ L*) (3)

where *k* is the permeability of the permeable part (m^2^), *A* is the average cross-section area of the permeable part (m^2^), *g* is the gravitational acceleration, Δ*h* is the hydrostatic gradient of the water in the bottle which is considered constant over time (m), *μ* is the dynamic viscosity of the water (Pa s), and *L* is the length of the permeable part (m).

For verification of the laminarity conditions, the Reynolds number was calculated according to the following definition [52]:

*Re* = (*ρ r v*)/*µ* (4)

where *r* is the average radius of the permeable part (m) and *v* is the flow speed of the water through the permeable part (m/s). All the Reynolds number values confirmed a condition of laminarity (Table 5).

By substituting Equation (3) into Equation (2) and integrating the ordinary differential equation, we obtain

*V* = (*ρ k A g* Δ*h t*)/(*µ L*) (5)

where *t* is the exposure time of the prototype in the soil. According to the simplifying conditions, this equation can fit the data in the initial conditions of the experiment, but it does not take into account the attenuation value due to soil saturation [53,54]. Equation (6) reports Equation (5), normalized with respect to the volume of water in saturated soil conditions:

*V*/*V_s_* = (*ρ k A g* Δ*h t*)/(*V_s_ µ L*). (6)

Equation (6) could be used for a linear fitting of the data at the origin of the axes from which the hydraulic permeability *k* was extrapolated. The model parameters are reported in Table 3.

## 3. Results and Discussion

### 3.1. Thermal Properties

**Figure 4:**
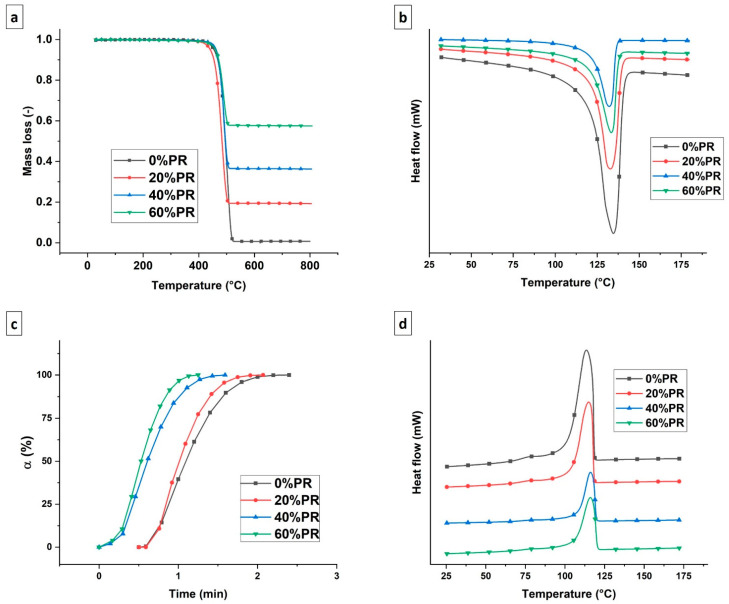
(**a**) Thermogravimetric analysis thermogram. (**b**) Differential scanning calorimetric second heating thermogram. (**c**) Relative crystallinity as a function of time. (**d**) Differential scanning calorimetric second cooling thermogram.

To investigate the effect of the pozzolan on the thermal properties of the studied composites, thermogravimetric analysis (TGA) and differential scanning calorimetric (DSC) analysis were performed. Figure 4a shows the TGA thermograms for all the composites as a function of the different percentages of pozzolan content. As can be observed, the decomposition kinetics were not perturbed significantly by the presence of fillers, maintaining the corresponding temperature to 50% of the mass loss around 500 °C. In other words, the thermal stability of the pure polymer used as a matrix was conserved. Figure 4b,d shows the second heating and cooling DSC thermograms, respectively. The curves depict insignificant differences in terms of the melting and cooling transitions having the same shape. However, the presence of the pozzolan reduced the melting enthalpy (Δ*H_m_*) and crystallization enthalpy (Δ*H_c_*) values. The numerical values are reported in Table 3. The possibility of melting the composites using less thermal energy can be an advantage for the extrusion of the material through the 3D printer nozzle, facilitating the phase transition of the polymeric filament in relation to the residence time of the material in the extruder [55]. Concerning crystallization kinetics, as is well known, the high-density polyethylene has fast crystallization kinetics [56]. Figure 4c shows the relative crystallization degree as a function of the time obtained at a constant temperature of 118 °C. Fast crystallization was observed for all the samples, and the half-crystallization time was in the range of 0.5–1.5 min. Meanwhile, there was a slight reduction in the degree of the crystallization after the pozzolan was added (Table 4), which was linked to the decrease in polymer chain mobility.

### 3.2. Rheological Properties

**Figure 5:**
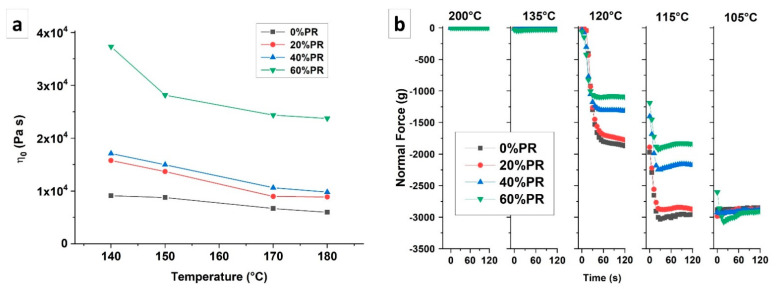
(**a**) Newtonian viscosity as a function of the rheological test temperature. (**b**) Normal force at a constant gap measured at different temperatures.

The rheological properties of the polymer composites were affected by the filler presence that interacted with the polymer matrix. In the case of highly filled composites for material extrusion process fabrication, the rheological behavior analysis had relevant importance to understanding the suitability of the material for the considered process. Figure 5a shows the evolution of the zero shear viscosity measured for samples at different temperatures (140 °C, 150 °C, 170 °C, and 180 °C). The results show that the zero shear viscosity increased by increasing the filler rate in the composites, leading to a reinforcing effect on the materials [57]. In particular, there was a slight increase in the viscosity for the composites at pozzolan rates of 20% and 40%, while a significant increase was observed for a pozzolan rate of 60%. This increase in viscosity that occurred in the case of the 60% pozzolan rate may indicate that the filler ratio was close to the higher maximum packing fraction [58]. In the meantime, the viscosity decreased linearly with the temperature (Figure 5a), except for the 60% composite, which had a faster reduction between 140 °C and 150 °C.

The gap test at a constant gap was used to analyze the effect of the pozzolan fillers on the volumetric contraction during the cooling of the composites. In particular, the test was performed by measuring the normal force evolution due to the temperature variation. The rheograms related to the normal force measurement are reported in Figure 5b, and the plotted curves are relative to the five temperatures chosen to focus on as the most important areas of the data results. The results show that the presence of the pozzolan slowed down the increase in normal force evolution for all the studied samples. In particular, at 120 °C and 115 °C, the normal force of the composites was lower compared with the pure polymer. A similar value of normal force was achieved for temperatures below 105 °C. Consequently, the presence of the pozzolan reduced the macromolecular mobility, limiting the phenomenon of volumetric shrinkage [59]. This behavior is interesting in light of the 3D printing process. In fact, having a slower volumetric reduction allows for greater cohesion between the deposited layers and greater geometric precision of the printed part, reducing the residual stress [60]. This analysis is in agreement with what was observed during the printing of the composite. Indeed, the composite materials showed a significant reduction in the warpage phenomenon and therefore better conservation of the geometry of the object compared with neat HDPE. Consequently, the detachment force from the printing plate decreased significantly by decreasing the normal force, resulting from the volumetric shrinkage, leading to the ease of printing pozzolan-based composites.

### 3.3. Mechanical Properties

**Figure 6:**
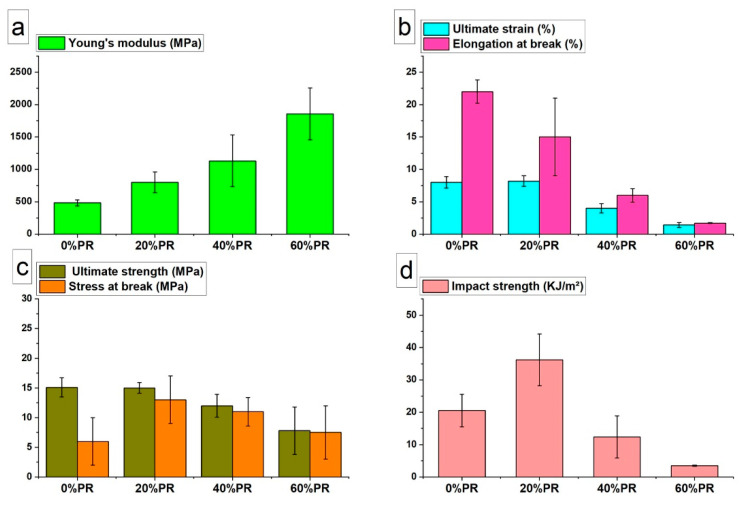
Tensile test results of 3D-printed specimens: (**a**) Young’s modulus, (**b**) ultimate strain and elongation at break, and (**c**) ultimate strength and stress at break. (**d**) Impact strength results from the impact test for 3D-printed specimens.

Figure 6 shows the tensile properties and impact strength values measured for the samples printed with a neat matrix and with the composites at 20%, 40%, and 60% pozzolan content. As can be observed in Figure 6a, Young’s modulus increased with the increase of the pozzolan content in the polymer matrix, highlighting a reinforcement of the matrix by the fillers. The ultimate strength and ultimate strain are reported in Figure 6b,c, respectively, and the results had the same value trends. In particular, the neat polymer samples and the composites samples at 20% pozzolan content did not present significant differences. However, by increasing the filler quantities, the elongation at break was reduced, and the maximum reduction was observed for the 60% pozzolan-filled composites (Figure 6b). This behavior is usually observed in polymeric composites, where the greater rigidity of the filler compared with the polymer matrix and the additional stress at the filler–polymer interface promote an increase in the elastic modulus of the composite. However, the presence of the filler generates phase heterogeneity and the discontinuity of the polymer matrix. Generally, this discontinuity translates into a lower ductility [61,62,63]. Concerning the stress at break (Figure 6c), an improvement of this property for all the composites was observed, showing a maximum value for the composites at 20% pozzolan content. The impact strength values measured for all the printed samples with different pozzolan content are shown in Figure 6d. As can be seen from the data, the trend was similar to the stress at break, where the maximum impact resistance value was read for the 20% pozzolan composite. Instead, the samples printed with 40% and 60% pozzolan-filled composites showed a reduction in impact resistance compared with the pure polymer. This unexpected result can be explained by considering that the composite at a filling ratio of 20% pozzolan mainly presented a reinforcing effect from the filler. However, with the increase in the filling ratio (40% and 60%), the discontinuity in the matrix generated by the pozzolan became important, reducing the stress at break and impact strength values [41,62]. Considering all the results, the optimal formulation was obtained for the composite with 20% pozzolan content.

These results are in accordance with the literature for the composites intended for 3D printing. For example, Kariz et al. (2018) [64] observed an improvement of 20% in the elastic modulus of polylactic-acid (PLA) by adding 50% (*w*/*w*) wood flour. Wu et al. (2017) [65] achieved, in a similar way, an improvement of 20% for polyhydroxyalcanoates (PHAs) with 40% (*w*/*w*) palm fiber in addition to a chemical compatibilizer. Concerning the literature studies on the BioPE-based composites, the maximum increase of the composites’ stiffness obtained by Tarrés et al. [24] was two times higher compared with neat BioPE. In this work, the maximum increase of the elastic modulus for the composites was three times higher than pure BioPE.

Another advantage in this work was the ability to print BioPE correctly with filler mass ratios up to 60% without any chemical treatments performed on the filler. In fact, in the majority of the cases, the maximum mass ratio added to (Bio)PE for 3D printing was around 30% [24,26,66].

### 3.4. Drip Watering Prototype Characterization

In this section, a prototype for precision drip irrigation is studied. In particular, the effect of the print fill density on the void morphology and water permeability are analyzed. For the printing of the prototypes, the optimal formulation obtained with the composite material, having a ratio of 20% pozzolan, is used.

#### 3.4.1. Morphological Analysis

**Figure 7:**
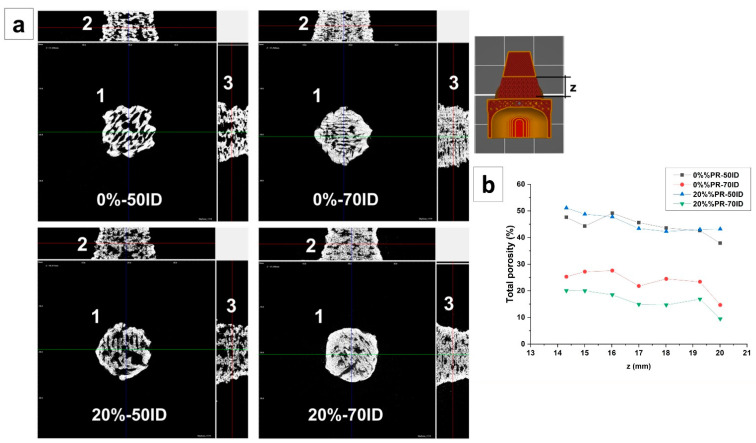
(**a**) Axonometric projection images analyzed with X-ray tomography. (**b**) Total porosity, measured along the permeable part of the prototype.

The morphological analysis of the permeable part of the drip watering prototype was carried out with X-ray tomography. Figure 7a shows the axonometric projection of the samples printed with the filament composites at 0% and 20% pozzolan and with different infill densities (50% and 70% ID). As can be seen from the images, the lattice structure had less microvoids between layers for the samples printed using a lower ID. However, at 70% ID, the 20% pozzolan composites demonstrated fewer microvoids between layers compared with the sample printed with a pure polymer. This behavior suggests that the pozzolan increases the adhesion between the layers of material deposited during printing. The total porosity values are reported in Figure 7b, quantifying this observation and showing the dependency of the porosity with respect to the height z of the permeable part. In particular, for each analyzed sample, the porosity was reduced with an increasing z value. This phenomenon was attributable to the greater cohesion of the material due to the reduction of the diameter of the piece as the height increased with the conical geometry, therefore leading to a smaller surface of the deposited layer. The smaller surface allowed a lower heat exchange time and therefore a lower cooling rate of the composite, permitting greater welding of the layers [47].

#### 3.4.2. Water Flow Measuring

The physical (working) principle of the system is described by a simplified example in Figure 3. In particular, (1) when the bottle is rotated, the water flows out due to the hydrostatic gradient of the liquid volume in the bottle. (2) However, the flow stops due to the vacuum effect that occurs in the close volume of air present in the bottle. (3) At this point, the bottle is placed in the soil, which will tend to absorb the water present in the pores of the prototype. (4) When the volume of water in the pores is such that it allows the passage of air due to the pressure difference between the outside and the inside of the bottle, the initial pressure of the gas phase in the bottle will be restored. Therefore, in the absence of a plant, the cycle will continue until the soil is saturated. Furthermore, the flow of water is strictly dependent on the quantity of microvoids distributed on the surface of the permeable part, which is the contact surface between the soil and the water.

This operating system can allow for adjusting the supply of water according to the needs of the plant by controlling the morphology of the prototype voids through the printing parameters and the choice of the material (e.g., ID or fillers).

**Figure 8:**
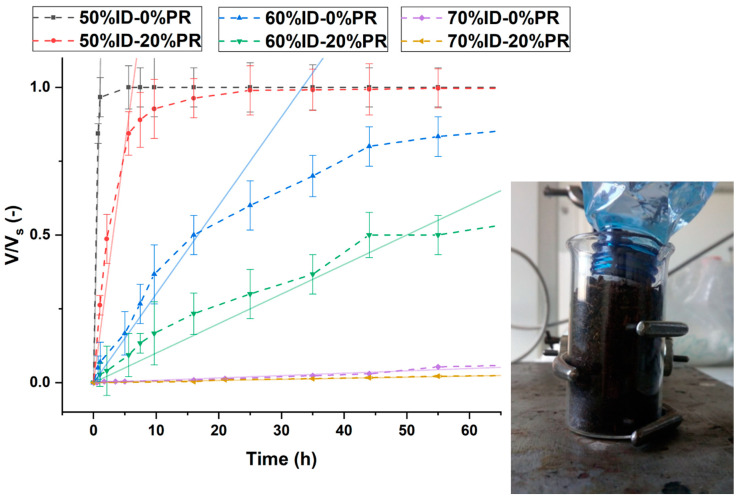
The volume of water absorbed and normalized on the sutured volume of the simulating soil as a function of time. The scatter curves show the data results, while the line curves show the model results.

Figure 8 shows the normalized water volume absorbed by the soil in the beaker at different times. A significant decrease in the water volume absorbed by the soil for the samples printed at 70% ID could be observed. The reduction of the microvoids passing from the lower to higher ID allowed lower water flow, with an order of magnitude in the range from ml/h to ml/day. However, the composites filled with 20% pozzolan had a slower watering rate compared with the pure HDPE. This result highlights that the pozzolan improved the adhesion between the layers and consequently reduced the microvoids, as was observed by X-ray tomography for the samples printed at 70% ID [41,67]. This observation was confirmed by the hydraulic permeability values (Table 5) calculated starting from the linear fitting of the kinetic model reported in Figure 8. The values showed a reduction of the permeability as the 3D printing ID increased and for the samples printed with filament composites.

## 4. Conclusions

In this study, a new polymeric composite filament based on high-density biopolyethylene and pozzolan by-product was produced and used for the material extrusion process. In particular, four formulations at 0%, 20%, 40% and 60% pozzolan were obtained. The composites did not present a difference in terms of thermal decomposition compared with the neat matrix, showing good thermal stability. The presence of the pozzolan decreased the melting enthalpy and the crystallinity degree. In terms of viscoelastic behavior, the Newtonian viscosity increased significantly for the composite at a 60% pozzolan content, reaching the maximum packing fraction. Concerning the normal force evolution, it was observed that the presence of the pozzolan limited the volumetric shrinkage during the cooling of the composites.

The printing of objects with complex geometries was successfully achieved. The printed objects with the composite’s filaments showed an increase in the elastic modulus, stress at break, and impact strength, but at the same time, the ultimate strain, elongation at break, and ultimate strength were reduced compared with the neat polymer. In other words, an improvement of the material rigidity with an optimal formulation at a 20% pozzolan ratio was observed. Finally, a drip watering prototype was conceived and printed with different infill densities to control the water flow of irrigation. As was shown by the X-ray tomography images, the pozzolan improved the cohesion between the layers of the final object, reducing the microvoids of the permeable part of the drip watering prototype.

From this study, it can be concluded that the huge amount of generated pozzolan by-products from quarries can be valorized as a filler for polymeric matrices, leading to the improvement of their properties. At the same time, it was demonstrated that the percentage of the pozzolan filler could reach up to 60% (*w*/*w*) for HDPE 3D printing filaments without any further treatment. Moreover, a decrease in the amount of the polymeric matrix used in the composites can lead to an important economic impact on the elaboration of new eco-friendly composites. Finally, some works are underway in our laboratory to develop pozzolan-based composites with a polymeric matrix that is fully biodegradable in the soil. An industrial application could be imagined as a seed pot directly implanted in the soil without further transplanting. After the matrix’s biodegradation, the pozzolan remaining in the soil can play a fertilizing role. This work will be a subject for future publication.

## Figures and Tables

**Table 1:** Extrusion conditions for production of the filaments.

Heating Zone	Z1	Z2	Z3	Z4	Z5	Z6	Z7	Z8
Temperature (°C)	180	220	220	220	230	240	240	220
Screw Rate (rpm)	200							
Total Mass Flow (kg/h)	0.6							

**Table 2:** 3D printing process parameters.

Parameter	Value
Nozzle diameter (mm)	0.6
Nozzle temperature (°C)	265
Layer thickness (mm)	0.15
Bed temperature (°C)	35
Printing speed for the first layer (mm/s)	20
Printing speed for the other layers (mm/s)	20

**Table 3:** Constant parameters used in the model.

Parameters	Values
*ρ* (kg/m^3^)	1000
*µ* (Pa s)	0.0001
*r* (mm)	5
*L* (mm)	7
Δ*h* (cm)	18
*V_s_* (cm^3^)	30

**Table 4:** Thermal properties obtained by differential scanning calorimetric analysis.

Pozzolan	Δ*H_m_* (J/g)	*T_m_* (°C)	Δ*H_c_* (J/g)	*T_c_* (°C)	*X_c_* (%)
0%	112	135	127	114	39
20%	74	133	89	115	33
40%	58	133	64	116	34
60%	36	134	43	116	34

**Table 5:** Results of the Reynolds number and hydraulic permeability of the permeable part of the prototype.

Samples	Reynolds Number	*k* (m^2^)
50%ID–0%PR	1.2 × 10^0^	3.2 × 10^−18^
50%ID–20%PR	2.7 × 10^−1^	5.3 × 10^−19^
60%ID–0%PR	3.7 × 10^−1^	9.9 × 10^−20^
60%ID–20%PR	2.1 × 10^−1^	3.3 × 10^−20^
70%ID–0%PR	2.5 × 10^−3^	2.6 × 10^−21^
70%ID–20%PR	7.1 × 10^−4^	1.2 × 10^−21^

## Data Availability

The data presented in this study are available on request from the corresponding author.

## References

[1] Crump S.S. (1992). Apparatus and Method for Creating Three-Dimensional Objects. U.S. Patent.

[2] Braconnier D.J., Jensen R.E., Peterson A.M. (2020). Processing Parameter Correlations in Material Extrusion Additive Manufacturing. Addit. Manuf..

[3] Chard J., van Iersel M., Bugbee B. (2010). Mini-Lysimeters to Monitor Transpiration and Control Drought Stress: System Design and Unique Applications.

[4] Hess L., De Kroon H. (2007). Effects of Rooting Volume and Nutrient Availability as an Alternative Explanation for Root Self/Non-Self Discrimination. J. Ecol..

[5] Schrader J.A., Kratsch H.A., Graves W.R. (2016). Bioplastic Container Cropping Systems: Green Technology for the Green Industry.

[6] Poorter H., Bühler J., van Dusschoten D., Climent J., Postma J.A. (2012). Pot Size Matters: A Meta-Analysis of the Effects of Rooting Volume on Plant Growth. Funct. Plant Biol..

[7] Ray J.D., Sinclair T.R. (1998). The Effect of Pot Size on Growth and Transpiration of Maize and Soybean during Water Deficit Stress. J. Exp. Bot..

[8] Biran I., Eliassaf A. (1980). The Effect of Container Size and Aeration Conditions on Growth of Roots and Canopy of Woody Plants. Sci. Hortic..

[9] Nikolaou G., Neocleous D., Katsoulas N., Kittas C. (2019). Irrigation of Greenhouse Crops. Horticulturae.

[10] Massetani F., Savini G., Neri D. (2014). Effect of Rooting Time, Pot Size and Fertigation Technique on Strawberry Plant Architecture. J. Berry Res..

[11] Ujeniya P.S., Rachchh N.V. (2019). A Review on Manufacturing, Machining, and Recycling of 3D Printed Composite Materials. IOP Conference Series: Materials Science and Engineering.

[12] Askanian H., Muranaka de Lima D., Commereuc S., Verney V. (2018). Toward a Better Understanding of the Fused Deposition Modeling Process: Comparison with Injection Molding. 3D Print. Addit. Manuf..

[13] Vidakis N., Petousis M., Tzounis L., Maniadi A., Velidakis E., Mountakis N., Kechagias J. (2021). Sustainable Additive Manufacturing: Mechanical Response of Polyamide 12 over Multiple Recycling Processes. Materials.

[14] Vidakis N., Vairis A., Petousis M., Savvakis K., Kechagias J. (2016). Fused Deposition Modelling Parts Tensile Strength Characterisation. Acad. J. Manuf. Eng..

[15] Kumar M., Ramachandran R., Omarbekova A. (2019). 3D Printed Polycarbonate Reinforced Acrylonitrile–Butadiene–Styrene Composites: Composition Effects on Mechanical Properties, Micro-Structure and Void Formation Study. J. Mech. Sci. Technol..

[16] Rodzeń K., Sharma P.K., McIlhagger A., Mokhtari M., Dave F., Tormey D., Sherlock R., Meenan B.J., Boyd A. (2021). The Direct 3D Printing of Functional PEEK/Hydroxyapatite Composites via a Fused Filament Fabrication Approach. Polymers.

[17] HDPE. https://polymerdatabase.com/Commercial%20Polymers/HDPE.html.

[18] Zander N.E., Gillan M., Burckhard Z., Gardea F. (2019). Recycled Polypropylene Blends as Novel 3D Printing Materials. Addit. Manuf..

[19] Sang L., Han S., Li Z., Yang X., Hou W. (2019). Development of Short Basalt Fiber Reinforced Polylactide Composites and Their Feasible Evaluation for 3D Printing Applications. Compos. Part B Eng..

[20] Lee J.-Y., An J., Chua C.K. (2017). Fundamentals and Applications of 3D Printing for Novel Materials. Appl. Mater. Today.

[21] Wang T.-M., Xi J.-T., Jin Y. (2007). A Model Research for Prototype Warp Deformation in the FDM Process. Int. J. Adv. Manuf. Technol..

[22] Plastics Market Size, Share & Trends Report, 2020–2027. https://www.grandviewresearch.com/industry-analysis/global-plastics-market.

[23] Schirmeister C.G., Hees T., Licht E.H., Mülhaupt R. (2019). 3D Printing of High Density Polyethylene by Fused Filament Fabrication. Addit. Manuf..

[24] Tarrés Q., Melbø J.K., Delgado-Aguilar M., Espinach F.X., Mutjé P., Chinga-Carrasco G. (2018). Bio-Polyethylene Reinforced with Thermomechanical Pulp Fibers: Mechanical and Micromechanical Characterization and Its Application in 3D-Printing by Fused Deposition Modelling. Compos. Part B Eng..

[25] Balani S.B., Chabert F., Nassiet V., Cantarel A. (2019). Influence of Printing Parameters on the Stability of Deposited Beads in Fused Filament Fabrication of Poly (Lactic) Acid. Addit. Manuf..

[26] Mazzanti V., Malagutti L., Mollica F. (2019). FDM 3D Printing of Polymers Containing Natural Fillers: A Review of Their Mechanical Properties. Polymers.

[27] Stoof D., Pickering K. (2018). Sustainable Composite Fused Deposition Modelling Filament Using Recycled Pre-Consumer Polypropylene. Compos. Part B Eng..

[28] Chong S., Pan G.-T., Khalid M., Yang T.C.-K., Hung S.-T., Huang C.-M. (2017). Physical Characterization and Pre-Assessment of Recycled High-Density Polyethylene as 3D Printing Material. J. Polym. Environ..

[29] Peng F., Jiang H., Woods A., Joo P., Amis E.J., Zacharia N.S., Vogt B.D. (2019). 3D Printing with Core–Shell Filaments Containing High or Low Density Polyethylene Shells. ACS Appl. Polym. Mater..

[30] Mohan V.B., Bhattacharyya D. (2020). Mechanical, Electrical and Thermal Performance of Hybrid Polyethylene-Graphene Nanoplatelets-Polypyrrole Composites: A Comparative Analysis of 3D Printed and Compression Molded Samples. Polym. Plast. Technol. Mater..

[31] Zhang F., Ma G., Tan Y. (2017). The Nozzle Structure Design and Analysis for Continuous Carbon Fiber Composite 3D Printing. Proceedings of the 2017 7th International Conference on Advanced Design and Manufacturing Engineering (ICADME 2017).

[32] Wang X., Jiang M., Zhou Z., Gou J., Hui D. (2017). 3D Printing of Polymer Matrix Composites: A Review and Prospective. Compos. Part B Eng..

[33] Naigaga E. (2014). An Examination of the Sustainability of Pozzolana Mining Processes in Uganda. Int. J. Res. Chem. Metall. Civ. Eng..

[34] El Youbi M.S., Ahmed E. (2017). Development and Study of Physical, Chemical and Mechanical Properties of a New Formulation of Cement of a Varying Percentage of Natural Pozzolan. J. Chem. Technol. Met..

[35] Kim H., Choi S., Lee B., Kim S., Kim H., Cho C., Cho D. (2007). Thermal Properties of Bio Flour-Filled Polypropylene Bio-Composites with Different Pozzolan Contents. J. Therm. Anal. Calorim..

[36] Sbaffoni S., Boni M.R., Vaccari M. (2015). Potential of Compost Mixed with Tuff and Pozzolana in Site Restoration. Waste Manag..

[37] Seco A., Ramirez F., Miqueleiz L., Urmeneta P., García B., Prieto E., Oroz V. (2012). Types of Waste for the Production of Pozzolanic Materials—A Review.

[38] Rocher P. (1992). Mémento Roches et Minéraux Industriels.

[39] Les Services de l’État dans le Puy-de-Dôme Schéma Départemental des Carriers. http://www.puy-de-dome.gouv.fr/schema-departemental-des-carrieres-r1292.html.

[40] Pouzzolane. http://www.ciment.wikibis.com/pouzzolane.php.

[41] Schiavone N., Verney V., Askanian H. (2021). Pozzolan Based 3D Printing Composites: From the Formulation Till the Final Application in the Precision Irrigation Field. Materials.

[42] Esposito Corcione C., Palumbo E., Masciullo A., Montagna F., Torricelli M.C. (2018). Fused Deposition Modeling (FDM): An Innovative Technique Aimed at Reusing Lecce Stone Waste for Industrial Design and Building Applications. Constr. Build. Mater..

[43] Wasti S., Adhikari S. (2020). Use of Biomaterials for 3D Printing by Fused Deposition Modeling Technique: A Review. Front. Chem..

[44] https://www.hitachi-hightech.com/file/global/pdf/products/science/appli/ana/thermal/application_TA_026e.pdf.

[45] Refaa Z., Boutaous M., Xin S., Siginer D.A. (2017). Thermophysical Analysis and Modeling of the Crystallization and Melting Behavior of PLA with Talc. J. Anal. Calorim..

[46] Marek A.A., Verney V. (2015). Rheological Behavior of Polyolefins during UV Irradiation at High Temperature as a Coupled Degradative Process. Eur. Polym. J..

[47] Schiavone N., Verney V., Askanian H. (2020). Effect of 3D Printing Temperature Profile on Polymer Materials Behavior. 3D Print. Addit. Manuf..

[48] Wypych G., Wypych G. (2018). 2-Mechanisms of Adhesion. Handbook of Adhesion Promoters.

[49] Graziano A., Jaffer S., Sain M. (2019). Review on Modification Strategies of Polyethylene/Polypropylene Immiscible Thermoplastic Polymer Blends for Enhancing Their Mechanical Behavior. J. Elastomers Plast..

[50] (2019). Perry’s Chemical Engineers’ Handbook.

[51] Whitaker S. (1986). Flow in Porous Media I: A Theoretical Derivation of Darcy’s Law. Transp. Porous Med..

[52] Bird R.B., Stewart W.E., Lightfoot E.N. (2006). Transport Phenomena.

[53] Ouedraogo F., Cherblanc F., Naon B., Bénet J.-C. (2013). Water Transfer in Soil at Low Water Content. Is the Local Equilibrium Assumption Still Appropriate?. J. Hydrol..

[54] (2016). Fundamental Equations in Colloid and Surface Science. Introduction to Applied Colloid and Surface Chemistry.

[55] Phan D.D., Horner J.S., Swain Z.R., Beris A.N., Mackay M.E. (2020). Computational Fluid Dynamics Simulation of the Melting Process in the Fused Filament Fabrication Additive Manufacturing Technique. Addit. Manuf..

[56] Patki R.P., Phillips P.J. (2008). Crystallization Kinetics of Linear Polyethylene: The Maximum in Crystal Growth Rate–Temperature Dependence. Eur. Polym. J..

[57] Valentina I., Haroutioun A., Fabrice L., Vincent V., Roberto P. (2018). Poly(Lactic Acid)-Based Nanobiocomposites with Modulated Degradation Rates. Materials.

[58] Escócio V.A., Pacheco E.B.A.V., da Silva A.L.N., de Paula Cavalcante A., Visconte L.L.Y. (2015). Rheological Behavior of Renewable Polyethylene (HDPE) Composites and Sponge Gourd (*Luffa cylindrica*) Residue. Int. J. Polym. Sci..

[59] Shah P., Stansbury J. (2014). Role of Filler and Functional Group Conversion in the Evolution of Properties in Polymeric Dental Restoratives. Dent. Mater..

[60] Casavola C., Cazzato A., Moramarco V., Pappalettera G. (2017). Residual Stress Measurement in Fused Deposition Modelling Parts. Polym. Test..

[61] Askanian H., Verney V., Commereuc S., Guyonnet R., Massardier V. (2015). Wood Polypropylene Composites Prepared by Thermally Modified Fibers at Two Extrusion Speeds: Mechanical and Viscoelastic Properties. Holzforschung.

[62] Kiran M.D., Govindaraju H.K., Jayaraju T., Kumar N. (2018). Review-Effect of Fillers on Mechanical Properties of Polymer Matrix Composites. Mater. Today: Proc..

[63] Jancar J., Friedrich K., Breuer U. (2015). Chapter 31—Composite materiomics: Multi length scale hierarchical composites for structural and tissue engineering applications. Multifunctionality of Polymer Composites.

[64] Kariz M., Sernek M., Obućina M., Kuzman M.K. (2018). Effect of Wood Content in FDM Filament on Properties of 3D Printed Parts. Mater. Today Commun..

[65] Wu C.-S., Liao H.-T., Cai Y.-X. (2017). Characterisation, Biodegradability and Application of Palm Fibre-Reinforced Polyhydroxyalkanoate Composites. Polym. Degrad. Stab..

[66] Filgueira D., Holmen S., Melbø J.K., Moldes D., Echtermeyer A.T., Chinga-Carrasco G. (2018). 3D Printable Filaments Made of Biobased Polyethylene Biocomposites. Polymers.

[67] Liao Y., Liu C., Coppola B., Barra G., Di Maio L., Incarnato L., Lafdi K. (2019). Effect of Porosity and Crystallinity on 3D Printed PLA Properties. Polymers.

